# Abstracts

**DOI:** 10.1155/S1463924685000141

**Published:** 1985


Journal of Automatic Chemistry/*Journal of Clinical Laboratory Automation,* Vol. 7, No. 2 (Apr–Jun 1985), pp. 60–63

### Abstracts

### Page 64

### An inexpensive fast memory module for rapid acquisition of digital data

D. T. Rossi and H. L. Pardue

This paper describes the design, construction, and performance of a modular memory system for rapid collection of digital data. The memory module can collect 64 kilobytes of data in as little as 64 ms. It has been used in conjunction with a diode array spectrophotometer to monitor the irreversible hydrolysis of p-benzoquinoneimine in an optically transparent thin layer electrochemical cell.

### Un module mémoire rapide et bon marché pour l'acquisition de données digitales

D. T. Rossi and H. L. Pardue

Cet article décrit les schémas, la construction et la capacité d'un système modulaire de mémoire pour une acquisition rapide de données digitales. Le module mémoire est capable de mémoriser 64 kilobytes de données en moins de 64 ms. Il a été utilisé avec un spectrophotomètre à diodes multiples qui surveille l'hydrolyse irréversible de p-benzodinonimène dans une cellule électrochimique optiquement transparente.

### Ein kostengünstiges, schnelles Speichermodul für die Erfassung digitaler Daten

D. T. Rossi and H. L. Pardue

Diese Arbeit beschreibt Entwurf, Konstruktion und Leistung eines modularen Speichersystems für die rasche Erfassung digitaler Daten. Das Speichermodul kann 64 Kilobyte Daten bis innerhalb von 64 Millisekunden abspeichern. Es wurde in Verbindung mit einem Multidioden-Spektrophotometer verwendet, das die irreversible Hydrolyse von p-Benzodinonimen in einer optisch transparenten elektrochemischen Zelle überwacht.

### Page 69

### The use of a microprocessor for flexible automation of an experimental procedure

M. S. Sheya and C. Riley

A microprocessor-controlled experimental procedure is described. A general-purpose program written for an Intel 8080 microprocessor was used to drive a stepper-motor/peristaltic-pump assembly. The program allows parameter pass for motor drive and control. With the aid of this program it was possible to automate the experimental procedure by running the motor in uni-step/multi-step mode, uni-rev/multi-rev mode and in a preprogrammed mode of operation. Facilities are also available in the program for varying the motor speed, repeating and aborting a task on-line. In this way the work of the experimenter was greatly simplified.

### L'utilisation d'un microprocesseur pour l'automatisation flexible d'une procédure expérimentale

M. S. Sheya and C. Riley

Une procédure expérimentale contrôlée par microprocesseur est décrite. Un programme écrit pour un microprocesseur Intel 8080 a été utilisé pour commander un moteur pas-à-pas combiné avec une pompe péristaltique. Le programme admet des paramètres pour la commande et le contrôle du moteur. Ce programme nous a permis d'automatiser une procédure expérimentale en utilisant le moteur dans les modes uni-step/multi-step, uni-rev/multi-rev et dans un mode programmé au préalable. Le programme prévoit aussi la possibilité de varier la vitesse du moteur et de répéter ou interrompre une procédure. De cette manière, le travail de l'expérimentateur est rendu beaucoup plus simple.

### Die Verwendung eines Mikroprozessors für die flexible Automatisierung eines experimentellen Ablaufs

M. S. Sheya and C. Riley

Es wird ein experimentelles Prozedere beschrieben, dass durch einen Mikroprozessor gesteuert wird. Ein allgemein verwendbares Programm, geschrieben für einen Intel 8080 Mikroprozessor, wurde benützt, um eine Kombination von Schrittmotor und peristaltischer Pumpe anzutreiben. Das Programm lässt Parameter zu für den Motorantrieb und -steuerung. Mit Hilfe dieses Programms war es möglich einen experimentellen Ablauf zu automatisieren, in dem der Motor in den Betriebsarten uni-step/multi-step, uni-rev/multi-rev und in einer vorprogrammierten Art benützt wurde. Das Programm enthält auch die Möglichkeit während des Betriebs die Motorgeschwindigkeit zu variieren und einen Ablauf zu wiederholen oder abzubrechen. Auf diese Art und Weise wird die Arbeit des Experimentators stark erleichtert.

### Page 74

### Application of statistical procedures in analytical instrument testing

W. Bablok and H. Passing

The evaluation of analytical instruments in clinical chemistry should provide comparable and reproducible data for users, manufacturers and officials. This means that as well as qualitative information, quantitative data is also needed to describe the performance of an instrument. The properties of main interest for instruments with continuous measurement values are precision, analytical range limits, carry-over, drift, accuracy, method comparison and interferences. Most of these items can be characterized by well-known descriptive statistics and only method comparisons require a procedure which allows for statistical inference.

For the biometrical evaluation of method comparison studies a number of different procedures are proposed. The assumptions under which these procedures were formulated, and their properties are discussed. Further, it is shown that the results depend on the appropriate sample size, the sampling distribution of the data and the precision of the instruments (methods). A recently published procedure, which is robust in respect of these requirements, is presented. Recommendations for the proper choice of the procedures are given.

**Application de méthodes statistiques pour l'évaluation d'instruments analytiques**
W. Bablok and H. Passing

L'évaluation d'instruments analytiques dans la chimie clinique devrait procurer des données comparables et reproducibles pour les utilisateurs, les fabricants et les autorités. Cela signifie que l'on utilise des informations qualitatives aussi bien que des données quantitatives pour décrire la capacité d'un instrument. Les propriétés principales d'instruments mesurant continuellement sont la précision, les limites de gamme analytique, le drift, l'exactitude, la comparaison de méthodes et interférences. La plupart de ces points peuvent être charactérisés par des statistiques descriptives connues, et seules les comparaisons de méthodes nécessitent une procédure qui considère les interférences statistiques.

Pour l'évaluation biométrique d'études de comparaison de méthodes un nombre de procédures différentes sont proposées. On discute les hypothèses sous lesquelles ces procédures et leurs propriétés ont été formulées. En outre, on prouve que les résultats dépendent d'une grandeur d'échantillon appropriée, de la répartition des échantillons et de la précision des instruments (méthodes). Une procédure publiée récemment qui répond à toutes exigences est présentée. Des recommandations pour le choix d'une procédure appropriée sont données.

**Applikation statistischer Methoden für das Austesten analytischer Instrumente**
W. Bablok and H. Passing

Die Evaluation analytischer Instrumente in der klinischen Chemie sollte in vergleichenbaren und reproduzierbaren Daten für Benützer, Hersteller und offizielle Stellen resultieren. Das bedeutet dass sowohl qualitative Informationen als auch quantitative Daten benötigt werden, die die Leistung eines Instruments beschreiben. Für Instrumente mit kontinurlicherier Messung gilt das Hauptinteresse den Eigenschaften Präzision, Grenzen des analytischen Bereichs, Verschleppung, Drift, Genauigkeit, Methodenvergleich und Interferenzen. Die meisten dieser Punkte können durch gut bekannte beschreibende Statistiken charakterisiert werden, und nur die Methodenvergleiche benötigen ein Prozedere, das statistische Interferenzen berücksichtigt.

Für die biometrische Auswertung von Methodenvergleichs-Studien werden eine Anzahl verschiedener Prozeduren vorgeschlagen. Die Annahmen, unter denen diese Prozeduren formuliert wurden, und deren Eigenschaften werden diskutiert. Ferner wird gezeigt, das die Resultate von einer geeigneten Probengrösse, Stichprobenverteilung der Daten und der Präzision der Instrumente (Methoden) abhängen. Ein kürzlich veröffentlichtes Prozedere, welches in Bezug auf diese Anforderungen robust ist, wird vorgestellt. Empfehlungen für die geeignete Wahl der Prozeduren werden angeführt.

---

**Page 80**

**A comprehensive laboratory automation system**
B. H. Venter and M. L. Siebert

A flexible, computerized data-processing system has been developed for handling results obtained in a routine chemical analytical laboratory that performs some 120 000 analyses per year. The results include about 80 different determinants while the number and type of samples, the combination of determinants, the frequency of samples submission and the time allowed for analyses vary constantly. In the past this service required time-consuming and cumbersome paperwork. With the new data-processing system results are fed directly from analytical instruments into the computer, which calculates the concentration, deviation and standard accuracy for each determinant, permanently stores all results, and produces data in the form of various statistical reports and graphs. The estimated 40% saving in labour, together with the benefits of data processing, data retrieval, quality control and automatic presentation of results in various convenient forms, fully justifies the high installation cost of the system.

**Un système d'automatisation de laboratoire à tâches multiples**

B. H. Venter and M. L. Siebert

Un système de traitement de données basé sur ordinateur a été développé pour traiter des résultats obtenus d'un laboratoire de chimie analytique de routine exécutant environ 120 000 analyses par an. Les résultats comprennent environ 80 déterminants différents alors que le nombre et le type d'échantillons, la combinations des déterminants, la fréquence d'arrivée des échantillons et le temps accordé pour l'analyse varient constamment. Auparavant, ce processus nécessitait beaucoup de temps et de travail ennuyeux. Avec ce nouveau système de traitement de données les résultats peuvent être transmis directement des instruments analytiques à l'ordinateur qui détermine la concentration, la déviation et la précision standard pour chaque déterminante, mémorise en permanence tous les résultats et met les données à disposition sous la forme de divers rapports statistiques et graphiques. On estime épargner ainsi 40% de temps de travail. Ceci ajouté à tous les avantages d'un système moderne d'acquisition et de traitement de données ainsi qu'un contrôle de qualité et une présentation automatique des résultats sous différentes formes justifient entièrement les coûts élevés pour l'installation de ce système.

### Ein umfassendes Laborautomatisierungssystem
B. H. Venter and M. L. Siebert

Für die Verarbeitung der Resultate, die in einem routine-chemisch-analytischne Laboratorium, das im Jahr ungefähr 120 000 Analysen durchführt, anfallen, wurde ein flexibles, computerisiertes Datenerfassungssystem entwickelt. Die Resultate umfassen ungefähr 80 verschiedene Determinanten, während die Zahl und die Art der Proben, die Kombination der Determinanten, die Häufigkeit des Probenanfalls und die für die Analyse zur Verfügung stehende Zeit ständig variieren. In der Vergangenheit war dies mit zeitraubenden und mühsamen Arbeiten verbunden. Mit dem neuen Datenerfassungssystem können Resultate direkt vom analytischen Gerät in den Rechner eingespiesen werden, der die Konzentration, Abweichung und Standardgenauigkeit für jede Determinante bestimmt, alle Resultate archiviert, und die Daten in der Form von verschiedenen statistischen Berichten und Graphiken zur Verfügung stellt. Die auf 40% geschätzten Einsparungen in Arbeitsstunden, zusammen mit den Vorteilen der Datenverarbeitung, Datenerfassung, Qualitätskontrolle und automatische Darstellung der Resultate auf verschiedenste, bequeme Art, rechtfertigt völlig die hohen Kosten für Beschaffung und Einrichtung dieses Systems.

---

### Page 85

### X-ray photon attenuation measurement as a technique for monitoring liquid composition
S. Everett and D. J. Malcolme-Lawes

The attenuation of 22.10 keV Ag Ka and 32.06 keV Ba Ka radiation by liquid mixtures in a 2 cm path-length flow-cell has been examined as a method of monitoring the compositions of binary liquid mixtures and solutions flowing through small diameter pipes. Mixtures studied include hexane/decane, aqueous ferrous sulphate solution, cream, silicone resin/white spirit, and ethanol/water mixtures.

### Mesure atténuation photon de rayons X comme téchnique de contrôle de mélanges liquides

S. Everett and D. J. Malcolme-Lawes

L'atténuation de la radiation de 22.10 keV Ag Ka et 32.06 keV Ba Ka par une mixture liquide dans une celle à flux de 2 cm de longueur a été examinée comme une méthode de contrôle de mixtures et solutions liquides binaires qui coulent par des tuyaux de petit diamètre. Les mixtures étudiées sont: héxane/décane, solution de sulfate ferreux, la crème, la resine silicone/alcool et éthanol/eau.

### Die Bestimmung der Röntgenstrahlphoten-Attenuation als Technik für die Ueberwachung der Zusammensetzung von Flüssigkeiten
S. Everett and D. J. Malcolme-Lawes

Die Attenuation von 22.10 keV Ag Ka und 32.06 keV Ba Ka Strahlen durch Flüssigkeiten in einer Durchflusszelle von 2 cm Weglänge wurde überprüft im Hinblick auf die Ueberwachung von Zusammensetzungen binärer Flüssigmischungen und Lösungen, die durch Röhren mit kleinen Querschnitt fliessen. Die untersuchten Mischungen umfassen Hexan/Dekan, wässrige Eisensulfat-Lösung, Rahm, Silikonharz/Weissgeist und Alkohol/Wasser-Mischungen.

---

### Page 90

### Assessment of the Eppendorf ERIS analyser
S. J. Callaghan *et al.*

The Eppendorf ERIS analyser was comprehensively evaluated with respect to specimen evaporation, cuvette integrity, photometer characteristics, imprecision and inaccuracy for glucose, cholesterol, triglycerides, iron, urate, aspartate aminotransferase and $\gamma$-glutamyltransferase. Comparison with routine methods also gave information on linearity and potential interferences.

The instrument proved to have acceptable performance characteristics and was reliable and simple to operate. Many desirable features made the analyser very suitable for general laboratory operation in the small- to medium-size laboratory and for more dedicated functions in the large laboratory.

### Evaluation de l'analyseur Eppendorf
S. J. Callaghan *et al.*

Analyseur Eppendorf ERIS a été évalué en ce qui concerne evaporation de spécimens, l'intégrité de la cuvette, charactéristiques du photomètre, imprécision et inexactitude pour glucose, cholestérol, triglycerides, der, acide urique, aspartate aminotransférase et y-glutamyltransférase. Des comparisons avec des méthodes de routine informaient sur la linéarité et des interférences potentielles. L'intrument démontrait des charactéristiques de performance acceptables, il était sûr et simple à opérer. De différentes propriétés désirable rendent l'analyseur très approprí pour l'opération de laboratoire générale dans un laboratoire de petite ou moyenne taille et pour des functions plus spécialisées dans un grand laboratoire.

### Beurteilung des Eppendorf-Analysengeräts
S. J. Callaghan *et al.*

Das Eppendorf ERIS Analysengerät wurde in Bezug auf Probenverdunstung, Küvettenintegrität, Verhalten des Fotometers, und Genauigkeit und Reproduzierbarkeit fur Glukose, Cholesterol, Triglyzeride, Eisen, Urat, Aminotransferase-Aspertat und -Glutamyltransferase umfassend geprüft. Ein Vergleich mit Routinemethoden ergab zusätazlich Daten über Linearität und mögliche Interferenzen. Das Gerät zeigte eine akzeptable Leistungscharakteristik und war zuverlässig und einfach zu bedienen. Viele gewünschte Eigenschaften machen des Analysengerät geeignet für die generelle Benützung im kleinen bis mittelgrossen Labor und für spezielle Funktionen im Grosslabor.

---

### Page 95

### Calculation error following sample dilution: a proposal for processing such specimens using a MUMPS program
T. G. Pellar *et al.*

High analyte values require dilution before analyses. It was discovered, in a random survey, that there was a 12% error rate associated with the calculation of the actual analyte value from the diluted value. The authors have written a MUMPS program designed to run on all terminals of the laboratory's computer system that processes these dilution calculations in a standard fashion using pre-entered serum pool (diluent) values. Each calculation is printed and an Activity Log of all calculations is maintained. There have been no errors in dilution calculations since the introduction of this program.

**Erreur de calcul après dilution d'échantillon: une proposition pour le traitement de tels spécimen de tels spécimens utilisant un programme MUMPS**

T. G. Pellar *et al.*

Des échantillons à valeurs élevées doivent être diluées avant l'analyse. Nous avons constaté, dans une étude avec choix au hasard qu'une erreur de 12 % était due en calcul de la valeur réelle à partir de la valeur diluée. Nous avons écrit un programme MUMPS destiné à fonctionner sur tous les terminaux de l'ordinateur du laboratoire qui traitent ces calculs de dilution d'une façon standard utilisant des valeurs serum pool introduits. Chaque calcul est imprimé et un contrôle d'avance des tous les calculs est maintenu. Depuis l'introduction de ce programme il n'y avait plus d'érreur de calcul de dilution.

**Rechnungsfehler nach Probenverdünnung: Ein Vorschlag für die Verarbeitung solcher Specimen unter Verwendung eines MUMPS-Programms**
T. G. Pellar *et al.*

Proben mit hohem Gehalt der zu bestimmenden Komponente bedingen von der Analyse eine Verdünnung. In einer statistisch angeordneten Ueberprüfung entdeckten wir, dass eine 12 %-Fehlerrate mit der Berechnung des ursprünglichen Gehalts aus dem Wert für die verdünnte Probe verbunden war. Wir haben ein MUMPS-Programm geschrieben, das auf allen Stationen unseres Laborrechnersystems läuft und das diese Verdünnungsberechnungen standardmässig unter Benützung von voreingegebenen Serumpool-(Verdünner) Werten durchführt. Jede Berechnung wird ausgedruckt und es wird ein Aktivitäten-Log aller Berechnungen geführt. Seit der Einführung dieses Programms wurden keine Fehler mehr bei den Verdünnungsberechnungen mehr festgestellt.

---

### Page 99

**Evaluation of an automated haemolytic method for the determination of ASO antibodies**
F. Parri and E. de Majo

The anti-Streptolysin O (ASO) titer was determined on 600 serum samples in order to assess the new Taso-tec/Taso-matic automatic system. The method is based on a property of the Streptolysin O (SO)—erythrocytes are not haemolyed when it is oxidized, but they are haemolysed when it is in a reduced form. The addition of a reducing agent, as a starting reagent for the haemolysis reaction after the binding of antigen and antibodies, allows the initial rate of haemolysis to be followed. The rate is proportional to the ASO titer of the sample. All procedure can be automatically performed with the Taso-tec reagent and the Taso-matic machine, which completes up to 17 serum kinetic determinations simultaneously in 30 min. The automatic system was compared with the reference Rantz and Randall method ($y = 22.23 + 0.95x$; $r = 0.96$) and the Aso-Quantum method ($y = 121.76 + 043x$; $r = 0.73$). The results show excellent correlation between the automatic method and the reference method.

**Evaluation d'une méthode hémolitique automatique pour la détermination des anticorps ASO**

F. Parri and E. de Majo

Le titre antistreptolysine O (ASO) a été déterminé dans 600 échantillons de sérum afin d'évaluer le nouveau système automatique Taso-tec/Taso-matic. La méthode se base sur le fait que la streptolysine O (SO) dans sa forme oxydée n'hémolyse pas les érythrocites, mais qu'elle les hémolyse dans sa forme réduite. L'ajoutage d'un agent réducteur comme réactif de départ pour la réaction d'hémolyse après la liaison d'antigènes et des anticorps permet de suivre la vitesse initiale de l'hémolyse. La vitesse est proportionnelle au titre ASO de l'échantillon. La procédure est réalisée automatiquement avec le réactif Taso-tec et la machine Taso-matic qui permet de compléter jusqu'à 17 déterminations cynétiques simultanément en 30 minutes. Ce système automatique a été comparé avec la méthode de référence de Rantz et Randall ($y = 22.23 + 0.95x$; $r = 0.96$) et avec la méthode Aso-Quantum ($y = 121.76 + 043x$; $r = 0.73$). Les résultats montrent l'excellente corrélation entre la méthode automatique et la méthode de référence.

**Auswertung einer automatisierten chemolytischen Methode für die Bestimmung von ASO-Antikörpern**

F. Parri and E. de Majo

Um das neue, automatische Taso-tec/Taso-matic System zu beurteilen, wurde der Anti-Streptolysin-O-Titer (ASO) mit 600 Serumproben bestimmt. Die Methode basiert auf einer Eigenschaft von Streptolysin-O (SO), dass nämlich in oxydierter Form Erythrozyten nicht hämolysiert werden, dass das aber in der reduzierten Form der Fall ist. Die Zugabe einer reduzierenden Substanz als Initialisierungsreagens für die Hämolysereaktion, nachdem sich Antigen und Antikörper gebunden haben, erlaubt die Verfolgung der anfänglichen Hämolysegeschwindigkeit. Die Geschwindigkeit ist dem ASO-Titer der Probe proportional. Das ganze Prozedere kann automatisiert werden mit dem Taso-tec-Reagens und dem Taso-matic-Gerät, welches innerhalb 30 Minuten bis zu 17 kinetische Serumbestimmungen gleichzeitig ausführt. Das automatische System wurde mit der Referenzmethode von Rantz und Randall ($y = 22.23 + 0.95x$; $r = 0.96$) und der Aso-Quantenmethode ($y = 121.76 + 043x$; $r = 0.73$) verglichen. Die Ergebnisse zeigen ausgezeichnete Korrelation zwischen der automatischen Methode und der Referenzmethode.

